# Into the Wild: GWAS Exploration of Non-coding RNAs

**DOI:** 10.3389/fcvm.2018.00181

**Published:** 2018-12-17

**Authors:** Hector Giral, Ulf Landmesser, Adelheid Kratzer

**Affiliations:** ^1^Department of Cardiology, Charité - Universitätsmedizin Berlin, Corporate Member of Freie Universität Berlin, Humboldt-Universität zu Berlin, and Berlin Institute of Health, Berlin, Germany; ^2^DZHK (German Centre for Cardiovascular Research), Partner Site Berlin, Berlin, Germany; ^3^Berlin Institute of Health (BIH), Berlin, Germany

**Keywords:** lncRNA, genetic variant, GWAS, coronary artery disease, cardiometabolic disorders

## Abstract

Genome-wide association studies (GWAS) have proven a fundamental tool to identify common variants associated to complex traits, thus contributing to unveil the genetic components of human disease. Besides, the advent of GWAS contributed to expose unexpected findings that urged to redefine the framework of population genetics. First, loci identified by GWAS had small effect sizes and could only explain a fraction of the predicted heritability of the traits under study. Second, the majority of GWAS hits mapped within non-coding regions (such as intergenic or intronic regions) where new functional RNA species (such as lncRNAs or circRNAs) have started to emerge. Bigger cohorts, meta-analysis and technical improvements in genotyping allowed identification of an increased number of genetic variants associated to coronary artery disease (CAD) and cardiometabolic traits. The challenge remains to infer causal mechanisms by which these variants influence cardiovascular disease development. A tendency to assign potential causal variants preferentially to coding genes close to lead variants contributed to disregard the role of non-coding elements. In recent years, in parallel to an increased knowledge of the non-coding genome, new studies started to characterize disease-associated variants located within non-coding RNA regions. The upcoming of databases integrating single-nucleotide polymorphisms (SNPs) and non-coding RNAs together with novel technologies will hopefully facilitate the discovery of causal non-coding variants associated to disease. This review attempts to summarize the current knowledge of genetic variation within non-coding regions with a focus on long non-coding RNAs that have widespread impact in cardiometabolic diseases.

In the dawn of the millennium, the first draft of the human genome represented a major milestone in the path to decipher the genetic component of human disease. Further refinement of the human genome by the 1,000 Genomes Project mapped over 88 million variants from 26 populations where ~20 million correspond to common (frequency >0.5%) single-nucleotide polymorphisms (SNPs), a coverage of >95% of all estimated human common SNPs (1, 2). Other consortia such as Encyclopedia of DNA Elements (ENCODE) (3, 4) and Functional Annotation of the Mammalian Genome (FANTOM) (5) contributed to the generation of a detailed atlas of DNA functional elements and transcriptional units uncovering that more than 80–90% of the human genome is transcribed and display some functionality (4). In this context, Genome-wide association studies (GWAS) emerged as a fundamental tool to define single nucleotide polymorphisms (SNPs) associated to complex human traits or diseases (6–10). With regard to cardiovascular disease, GWAS studies identified up to 161 genetic risk loci associated to coronary artery disease (CAD) (11–13).

Despite the profound contributions of GWAS to the understanding of human disease pathophysiology, some issues forced to redefine the framework of GWAS studies. First, most significant GWAS hits could only explain a small fraction of genetic variance for a specific trait (14). In the case of CAD, all 161 genome-wide significant loci account for 15.1% of the predicted genetic contribution to the disease (15), which is strikingly similar to the percentage of gene sets (13.9%) or gene networks (14%) implicated on these 161 CAD-associated loci (12). An emerging notion, known as omnigenic model, states that cell regulatory networks are so deeply connected that basically all genes expressed in disease-relevant cell types conspire to influence the heritability of complex traits (16). Therefore, this model assumes that thousands of loci with small size effects contribute to the overall heritability of the trait or disease by affecting the expression of a smaller set of core genes (16). It seems that the common disease-common variant (CD-CV) model that drove the first decade of GWAS studies is shifting to a complex trait-complex genetics (CT-CG) scenario, where a handful of relevant variants cannot fully explain genetic variation in whole populations. The overall notion of a widespread dispersion of genetic contributions to disease due to the interconnectivity of biological systems seems to be widely accepted. On the other hand, the concept of a set of core genes driving the phenotype of complex diseases is still controversial and as a result the choice of methodology to address the future of the field (17).

Nearly 90% of all phenotype-associated SNPs identified by GWAS lied within non-coding regions (18–20), which includes a broad spectrum of locations including intronic or promoter regions, small ncRNAs such as miRNAs, long ncRNAs, antisense, and enhancer or insulator regions. Most non-coding variants are concentrated in deoxyribonuclease I (DNase I) hypersensitive sites that label regions with increased chromatin accessibility. Currently, around 2,500 miRNAs and more than 50,000 lncRNAs have been annotated in the human genome, practically doubling the number of protein coding transcripts, highlighting the important role of this part of the genome (21).

This review summarizes genetic variations within lncRNAs associated to cardiovascular disease (CAD, MI) and to various cardiometabolic risk factors for cardiovascular disease such as lipoprotein metabolism, diabetes or hypertension (Table 1).

**Table 1:** List of representative lncRNA and variants identified by GWAS associated to cardiometabolic disease traits.

**lncRNA gencode ID (Aliases)^**a**^**	**Cytogenetic region**	**Genes in locus**	**SNP variant^**b**^**	**Disease or phenotype traits**	**NHGRI Catalog^**c**^**	**Other sources^**d**^**	**GTEx eQTLs^**e**^**
**CDKN2B-AS1** ENSG00000240498 (ANRIL)	9p21.3 3.4kb 3' CDKN2B	CDKN2A, CDKN2B, DMRTA1	rs2891168 (intronic)	myocardial infarction CAD	GCST003117 ^(13)^ 5 × 10^−75^ GCST005194 ^(12)^ 5 × 10^−204^		none
	9p21.3p 13kb 3' CDKN2B	CDKN2A,CDKN2B, DMRTA1	rs4977574 (intronic) rs10811661 (intergenic)	CAD Type 2 Diabetes	GCST005195 ^(12)^ 9 × 10^−223^ GCST004894 ^(48)^ 2 × 10^−68^		none none
**MIAT** ENSG00000225783 (lnc-CRYBA4)	22q12.1		rs2331291 (intronic)	myocardial infarction		GWASdb v2 ^(63)^ 5 × 10^−7^	MIAT (10hits)
**H19** ENSG00000130600 (LINC00008)	11p15.5	HOTS, MRPL23	rs217727 (exonic)	Systolic blood pressure CAD	GCST006188 ^(72)^ 7 × 10^−15^	Meta-analysis ^(71)^ 1 × 10^−7^ Case-control genotyping ^(70)^ 1 × 10^−3^	
**LOC157273** ENSG00000248538 (AC022784.1; RP11-10A14.4)	8p23.1	PPP1RB	rs9987289 (intronic) rs4841132 (exonic)	HDL cholesterol Coronary artery calcification Fasting blood insulin	GCST002223 ^(75)^ 2 × 10^−44^ GCST001639 ^(77)^ 2 × 10^−9^ GCST005185 ^(76)^ 6 × 10^−15^		none none
**KCNQ1OT1** ENSG00000269821 (KCNQ1-AS2)	11p15.5	KCNQ1	rs231362 (exonic)	Type 2 Diabetes	GCST000712 ^(101)^ 3 × 10^−13^		none
**LINC00243** ENST00000419357 (lnc-IER3)	6p21.33		rs886424 (exonic)	Type 1 Diabetes Total cholesterol Total TG	GCST002876 ^(100)^ 3 × 10^−14^	Meta-analysis ^(29)^ 2 × 10^−9^ 8 × 10^−11^	(IER3, HLAs…) 153 hits
**LINC02137** ENSG00000260186 (RP11-481J2.2)	16q21		rs4784934 (intronic)	QT Interval	GCST002500 ^(82)^ 6 × 10^−9^		LINC02137 (4 hits) NDRG4 (1 hit)
**LOC400684** ENSG00000267213	19q13.11	ZNF507	rs12976411 (intronic)	CAD	GCST003116 ^(13)^ 1 × 10^−14^		ZNF507 (1hit)
**LINC00310** ENSG00000227456	21q22.11	MRPS6,SLC5A3	rs28451064 (intronic)	CAD myocardial infarction	GCST005195 ^(12)^ 1 × 10^−33^ GCST003117 ^(13)^ 6 × 10^−12^		none

*A profile of the well-studied lncRNAs such as ANRIL as well as other candidate lncRNAs that carry variants which have been either associated to CAD, MI or cardiometabolic traits which are high risk factors for the disease such as T2D, hypertension, obesity*.

a Official name; Gencode ID (GrCh38.p12); aliases

b Official variant ID; variant location in the locus

c NHGRI GWAS Catalog study ID described; reference of the study; p-value

d alternative sources such as GWASdb catalog or meta-analysis studies; reference of the study; p-value

e *GTEx eQTLs; genes associated to the variant (number of hits in the database)*.

## Impact of Genetic Variants on lncRNAs Functionality

One of the longest-standing challenges in human genetics is to assign potential causality within a locus to every variant in close linkage disequilibrium (LD) with the lead variant (34). Despite the potential of lncRNAs as causal factors of disease, GWAS studies had a tendency to explore genetic variant causality preferentially in coding genes, mostly due to our limited knowledge of ncRNAs genomic structure and functionality. Additionally, lncRNAs overlapping coding genes (such as antisense and intronic lncRNAs) are harder to dissociate from neighboring coding genes when searching for potential causal variants compared to intergenic lncRNA (lincRNA) which do not overlap coding genes. Fortunately, interactive lncRNA databases (LincSNP2.0) (35) together with established GWAS catalogs like NHGRI-EBI (36) and GWASdb.v2 (37) have started to integrate newly identified lncRNAs transcripts and disease-associated genetic variants. The latest databases mapped 371,647 disease-associated SNPs to lncRNA what accounts for approximately 45% of all disease-associated human SNPs identified (35).

Recent approaches focused on lincRNAs by further exploring loci previously associated to CAD (32, 38–41). For example, a class-level testing framework, termed Genetic Class Association Testing (GenCAT) allowed the identification of new trait-associated variants within multiple lincRNAs contributing novel insights into their role in cardiometabolic pathophysiology (42). GenCAT approach includes SNPs directly within the lincRNA but also the ones 500 kb up- or downstream of the lincRNA (38).

In a functional perspective, many lncRNAs reside in the nucleus conducting key regulatory steps in gene transcription, transcript splicing or chromatin structure. Cytoplasmic lncRNAs affect cell homeostasis by modulating translation and stability of mRNA through scaffolding multi-protein complexes that accomplish these functions (43). Several lncRNA functions depend on structural domains that generate binding sites to interact with RNA binding proteins (RBPs) acting as scaffolds for recruitment of proteins, RNA molecules and DNA elements (44–46). Some genetic variants are predicted to impact lncRNA secondary structure and thereby lncRNA–RBP interactions which can dramatically affect their functionality. Low evolutionary conservation of lncRNAs constitutes a challenge to predict structural domains and consequently how genetic variants induce functional modifications (47). Moreover, analysis of variation frequencies suggested that functional elements in lncRNAs have a much lower variation frequency almost comparable to protein-coding exons (48). Alternative splicing is an additional mechanism to generate functional diversity of lncRNAs by differential arrangement of structural domains (19).

Furthermore, SNPs may affect lncRNA transcriptional expression by altering its promoter region but also may influence expression of proximal or distal protein coding genes through the action of enhancers (19). Modulation of distant genes by trans-regulation is mediated by lncRNAs-enhancers but the effect of induced chromatin structural changes must be also considered. Chromatin structural loops link regulatory enhancer elements to distant gene promoters and variants disrupting this process broadly influence gene expression (49). Distal regulatory elements (DRE) can regulate the transcription of lincRNA through chromatin interactions, which can be influenced by GWAS-identified SNPs and define disease association (50).

## Long non-coding RNAs Associated to Cardiometabolic Traits

The first examples of SNP variants associated to increased risk of CAD located within a lncRNA were identified in the locus chr9p21.3, which resulted to be the CAD risk locus with the strongest effect found up to date. Locus chr9p21.3 contained multiple SNP variants at the antisense noncoding RNA in the INK4 locus (ANRIL), now referred to as CDKN2B-AS1 (51–53). CDKN2B-AS1 spans 126.3 kb in a gene cluster next to three tumor suppressor genes (p15/CDKN2B, p16/CDKN2A and p14/ARF), partially overlapping CDKN2B (53–55). Several CDKN2B-AS1 SNP variants also associated to other disease traits such as ischemic stroke, aortic aneurysm, atherosclerosis, specific carcinomas and type 2 diabetes (T2D) (22, 56–58).

**Figure 1:**
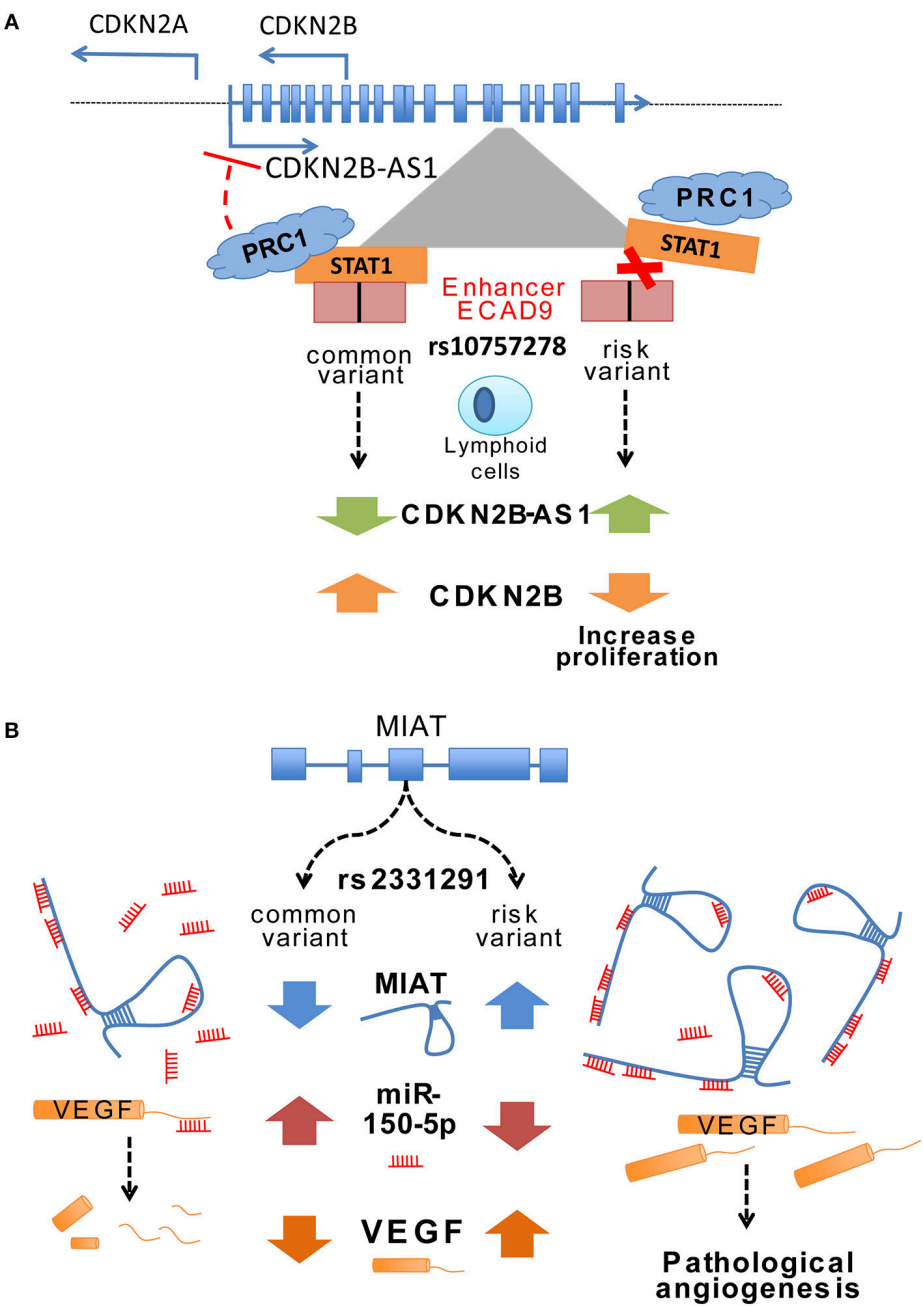
**(A)** Change of ANRIL expression through a variant in an enhancer region. The CAD associated variant rs107577278 lies within the binding site for the STAT1 transcription factor of enhancer region ECAD9. In lymphoid cells the binding of STAT1 to this region has been associated to decreased ANRIL expression, whereas silencing of STAT1 lead to an enhanced expression of ANRIL. The risk variant of rs107577278 disrupts the binding of STAT1 and the repression of ANRIL expression is abrogated. Increased expression of ANRIL promotes a downregulation of CDKN2B/p15 gene expression and underlines a proliferative effect which presumably increases CVD susceptibility. **(B)** Potential regulatory mechanisms of MIAT expression through different variants. Ishii et al. (63) unraveled that various variants are present in the lincRNA MIAT and associated them to myocardial infarction such as rs3132291. Some variants in Exon 5 have been associated to increased MIAT expression. Yan et al. showed in their study that MIAT can bind miR150-5p in endothelial cells and does inhibiting the degradation of its direct target VEGF. These data suggest that certain variants in the MIAT lincRNA can modify the structure of MIAT and thus leading to increased binding of miR-150-5p and consequently inhibiting the degradation of its target genes such as VEGF.

Most SNPs in the core risk region for CAD located within CDKN2B-AS1 intronic areas (118 out of 131 variants) where several enhancers were described (59). These enhancers mediated cys-regulation of neighboring genes like CDKN2A/B or methyl-thioadenosine phosphorylase (MTAP) but also trans-regulation of genes such as interferon-α21 (IFNA21), one million base pairs upstream (59). CDKN2B-AS1 trans-regulation of gene expression increased cell adhesion and proliferation, both atherogenic processes, in a process partially mediated by ALU elements located in CDKN2B-AS1 (60). Interestingly, CDKN2B-AS1 interacted with a component of the polycomb repressor complex (PRC) 1 and 2, which control the epigenetic repression of the CDKN2B gene (61, 62). In fact, risk variant rs10757278 located at enhancer ECAD9 inside CDKN2B-AS disrupted the binding site of STAT1 transcription factor (59). In lymphoid cells, this disruption of STAT1 binding implied a failure to recruit the repressor machinery and resulted in increased CDKN2B-AS expression, a mechanism that was confirmed by the silencing of STAT1 (Figure 1A) (59).

Only five of the CAD candidate variants are located in exons of CDKN2B-AS1 but none of them are located in conserved elements, questioning the likeliness to affect functional domains (59). However, numerous splice isoforms have been identified for CDKN2B-AS1 (14 isoforms, Genbank; 21 isoforms, GENCODE) highlighting a complex alternative splicing regulation that potentially affects the structural domain organization of the lncRNA leading to modulation of its functionality (64). Carriers of risk haplotype presented increased expression of CDKN2B-AS1 splice-isoforms EU741058 (short form) and NR_003529 (long form) but not DQ485454 (short form) which directly correlated with the severity of atherosclerosis, suggesting distinct roles for CDKN2B-AS1 splicing variants (65). Additionally, splicing isoforms defined by their polyadenylation site in proximal (exon 13) or distal (exon 19) showed trans-regulation of different set of genes. Proximal CDKN2B-AS1 isoforms modulated expression of glucose and lipid metabolism genes (66) while distal isoforms regulated RBMS1 (RNA Binding Motif Single Stranded Interacting Protein 1), a cell cycle suppressor (67). Conversely, circularized CDKN2B-AS1, another form of alternative splicing, showed an atheroprotective role via interaction with pescadillo homolog 1 (PES1) which leads to impaired ribosomal biogenesis (68). An SNP located in the 3′ region of CDKN2B-AS1 associated with reduced expression of CDKN2A, CDKN2B and CDKN2B-AS1 but also with increased VSMC proliferation (69). Other CDKN2B-AS1 variants confer increased myocardial infarction (MI) risk (70), supporting previous findings, where the level of CDKN2B-AS1 significantly increased in peripheral blood mononuclear cells after MI (71). Despite great efforts, causal mechanisms of CDKN2B-AS1 variants have been elusive and not fully unravel yet. For further detail, we refer the reader to other excellent recent reviews on the topic (23, 53, 72, 73).

Myocardial infarction associated transcript (MIAT) was identified as a susceptible locus for MI in a Japanese population by large-scale case-control associated study (63). MIAT expression upregulation in a MI mouse model concomitant with increased cardiac interstitial fibrosis suggested a profibrotic role with a prominent impact in the MI pathogenesis (74). Furthermore, *ex-vivo* experiments with a diabetic rat model identified a regulatory feedback loop between MIAT, vascular endothelial growth factor (VEGF) and miR-150-5p. MIAT acts as a sponge for miR-150-5p and represses degradation of VEGF mediated by miR-150-5p (Figure 1B) (75). Expression of both MIAT and CDKN2B-AS1 increased in human atherosclerotic arteries suggesting a potential role of MIAT on atherosclerotic plaque development (76).

The embryonic lincRNA H19 was identified to be re-expressed in human atherosclerotic plaques and in a rat model of carotid artery injury (77, 78). Recently, a genotyping study of 4 SNPs in H19 locus demonstrated significant association with CAD in a Chinese population (26). Additional GWAS and meta-analysis studies proved association of H19 variants with blood pressure, a well-known risk factor for cardiovascular disease (24, 25). Mechanistically, H19 was proposed to modulate availability of several let-7 miRNAs by acting as a molecular sponge (79). Highly expressed in adult muscle tissue, H19 modulation of let-7 likely controls timing of muscle differentiation since H19 depletion accelerates *in vitro* muscle differentiation with a concomitant overexpression of let-7 (79). Additionally, H19 was highly up-regulated in two different mouse models of abdominal aortic aneurism whereas specific H19 knock-down limited aneurism growth by a mechanism involving decreased apoptosis of smooth muscle cells (80). Other lncRNAs that contained genetic variants associated to CAD have been identified by GWAS studies but not studied further on their putative causal mechanisms such as LOC400684 an uncharacterized antisense RNA in the Zinc Finger Protein 507 (ZNF507) locus (12) or lncRNA LINC00310 which variant rs28451064 is also associated to myocardial infarction (13).

Genome-wide analysis also revealed multiple variants associated to cardiometabolic traits such as cholesterol levels or type 2 diabetes (T2D), both of them established risk factors of cardiovascular disease. For example, genetic variant lying in the lincRNA LOC157273 associated to lipid (HDL cholesterol) (27) and glycemic (fasting insulin levels) (29) traits but also to coronary artery calcification (28). Genetic variants at LOC157273 associated to expression changes of the nearby gene PPP1R3B, a phosphatase involved in hepatic regulation of glucose (81). Another SNP (rs886424) located in the second exon of LINC00243 associated with total cholesterol and triglyceride levels (32). Expression quantitative trait loci (eQTL) analysis also associated variant rs886424 with LINC00243 expression levels of as well as numerous nearby immune-related genes including immediate early response 3 (IER3) and several HLA forms (32). IER3 was reported to inhibit pro-inflammatory cytokines but the exact role of LINC00243 in immune-function and its putative link to cardiometabolic diseases requires further evaluation. One of the SNPs associated to T2D (rs231362) in the KCNQ1 locus overlaps both KCNQ1OT1 lncRNA antisense and the intron 11 of KCNQ1 (32). Several other polymorphisms in KCNQ1 locus associated also with cardiovascular events (82) and some showed protective effect against arrhythmic risk in long-QT syndrome (83). Both KCNQ1OT1 and CDKN2B-AS1 were shown to be valid predictors of left ventricle dysfunction after an MI (71). KCNQ1OT1 is an imprinted gene that is expressed only from the paternal allele and responsible to silence a proximal cluster of genes (84). Mechanistically, KCNQ1OT1 acts as a scaffold for the chromatin modifiers HMT G9a and PRC2 as well as DNA methyltransferase Dnmt1 which exerts gene repression by histone modifications and DNA methylation, respectively (84).

Finally, the ARIC (Atherosclerosis Risk in Communities) study intended to establish genetic loci associated to ECG global electrical heterogeneity (GEH) and consequently changes in QT measurements and one of the identified loci contained the lncRNA LINC02137 (33). LINC02137 was highly expressed in human heart atrial-appendage region and eQTL analysis showed that variant rs4784934 significantly associated with the expression of LINC02137 and gene NDRG4 in atrial tissue. NDRG4 was reported to be necessary for sodium channel trafficking in the nervous system but also associated with cardiomyopathy (85).

## Future Perspectives of lncRNA Genetic Variants

Determination of potential causality among genetic variants associated with cardiovascular and cardiometabolic diseases remains a challenging future task. In the case of the functional analysis of lncRNAs it is important to consider their low expression levels and high degree of tissue and cell type specificity. For example, tissue-specific expression quantitative trait loci (eQTL) analysis of lncRNAs is a strong tool to associate certain variants to downstream effectors. Genotype-Tissue Expression (GTEx) project provides the possibility to study tissue-specific gene expression and regulation on large scale with 44 various tissues in 449 individuals, which allowed to build up a resourceful platform in order to identify genetic associations both for local (cis eQTLs) and distal (trans eQTLs) effects (86). Nonetheless, it is relevant to indicate some limitations inherent to this analysis tool such as the inability to detect small size effect eQTLs due to multiple test burden, or the fact that eQTL effects are strongly tissue specific which hinders the inference of functionality and therefore caution must be taken to extrapolate conclusions to other tissues.

Novel lncRNA were localized near leukocyte enhancers and close to GWAS identified risk variants for autoimmune diseases suggesting alterations in enhancers or super enhancers might be associated to changes in phenotype and disease risk (87). SNP in close proximity or even in far distance (e.g., in trans location to the variant), may help unravel the complex regulatory events of cardiovascular disease including underlying importance of enhancers or super-enhancers (88). Yet, the term “super-enhancer” is under debate since a clear definition has not been established and their functional properties do not necessarily set them apart from regular enhancers (89). Another task for future studies is to determine the role of lncRNAs and their genetic variants in the maintenance and remodeling of the chromatin structure that drives interactions between enhancers and transcription initiation sites. Chromosome Conformation Capture (C3) technologies such as HiC (90, 91) or chromatin interaction analysis by paired-end tag sequencing (ChIA-PET) (92) will be useful as genome-wide approaches to study chromatin structural changes and to define the impact of genetic variants in long-range chromatin interactomes.

The advent of new sequencing technologies that improve current throughput, length of reads and cost will increase the number of annotated lncRNAs and help to define their complex transcript models. One of such technologies is capture long-read sequencing (CLS), a technique that uses lncRNA capture enrichment with nanopore technology, which allows sequencing of longer fragments (~1.5 kb) for characterizing the lncRNA structure (93). This highly promising approach would greatly improve the task of defining exon connectivity and therefore splicing transcript models.

Another feature to improve is our ability to predict and characterize lncRNA structural motifs and their underlying functional domains. Computational analysis approaches are able to predict the formation of loops and simple helices but are not so successful to define more complex motifs (94). New high-throughput techniques based on new generation sequencing (NGS) technologies emerged to define new motifs and validate computational predictions in a genome-wide scale (94). These methods use diverse RNA nucleases (ssRNA or dsRNA) or chemical probes in combination with NGS to analyze full transcriptomes in techniques such as Parallel Analysis of RNA Structure (PARS) (95), Fragmentation Sequencing (96) or Selective 2′ hydroxyl acylation analyzed by primer extension (SHAPE) (97, 98). For a detailed functional characterization of lncRNAs, novel identify structural domains should be linked to interactome information that can be obtained with novel technologies such as ChIRP (99) and CHART (100). These techniques allow the identification of specific lncRNA interacting partners such as RBPs and can also delimit the interaction sites to specific domains within the RNA molecule.

Lastly, it will be relevant to understand the potential regulatory effects that genetic variants within lncRNA have on regulation of CpG islands in cardiometabolic disorders (32). In fact, an integrative analysis of 11 human data sets generated a reference human epigenome as a framework to characterize GWAS variants that alter the epigenomic profile during complex human diseases (101), which can be also used to profile the non-coding genome.

In summary, in the post-GWAS era many relevant factors must be considered in order to study the effect of genetic variation in lncRNA, some of which comprise differential tissue expression, splicing isoforms models, RNA structural prediction and functional domain identification, and identification of lncRNA interacting partners such as RBPs. The high proportion of disease-associated SNPs lying in non-coding regions highlighted their functional relevance and prompted a better understanding of lncRNA biology as well as regulatory regions such as enhancer to unravel their potential role in cardiometabolic diseases. The expansion of the GWAS field to explore the functionality of lncRNA but also other non-coding RNAs will provide potential novel regulatory causal mechanisms of cardiovascular disease. This research area warrants interesting new insights into underlying mechanisms that determine the genetic component of human disease and will clear the path toward a personalized medicine approach.

## Author Contributions

AK and HG screened the literature on the topic, drafted, wrote and revised the article. UL revised the article.

### Conflict of Interest Statement

The authors declare that the research was conducted in the absence of any commercial or financial relationships that could be construed as a potential conflict of interest. The reviewer AS declared a shared affiliation, with no collaboration, with the authors to the handling Editor.
